# The electronic, structural and magnetic properties of La_1−1/3_Sr_1/3_MnO_3_ film with oxygen vacancy: a first principles investigation

**DOI:** 10.1038/srep22422

**Published:** 2016-03-01

**Authors:** Jia Li

**Affiliations:** 1 School of Science, Hebei University of Technology, Tianjin, 300401, People’s Republic of China

## Abstract

We have systematically investigated the influence of oxygen vacancy defects on the structural, electronic and magnetic properties of La_1−*x*_Sr_*x*_MnO_3_ (*x* = 1/3) film by means of ab initio calculations using bare GGA as well as GGA+U formalism, in the latter of which, the on-site Coulombic repulsion parameter U for Mn 3d orbital has been determined by the linear response theory. It is revealed that the introduction of the vacancy defects causes prominent structural changes including the distortion of MnO_6_ octahedra and local structural deformation surrounding the oxygen vacancy. The GGA+U formalism yields a significantly larger structural change than the bare GGA method, surprisingly in contrast with the general notion that the inclusion of Hubbard U parameter exerts little influence on structural properties. The distortion of MnO_6_ octahedra leads to a corresponding variation in the hybridization between Mn 3d and O 2p, which gets strengthened if the Mn-O distance becomes smaller and vice versa. The magnetic moments of the Mn atoms located in three typical sites of the vacancy-containing supercell are all larger than those in the pristine system. We have characterized the O-vacancy defect as a hole-type defect that forms a negative charge center, attracting electrons.

The perovskite manganese oxides La_1−*x*_A_*x*_MnO_3_ (A = Ca, Ba, Sr) have attracted extensive attention thanks to their special electronic and magnetic properties as well as their potential applications^1^,^2^,^3^. Among this group of the materials, La_1−*x*_Sr_*x*_MnO_3_ (LSMO) exhibits exotic properties such as colossal magnetoresistance (CMR) and metal-insulator transition^4^, which originate from the delicate coupling between charge, spin, and orbital and lattice degrees of freedom. The LSMO films, in particular, have the potential for commercial applications owing a variety of unique magnetic and magnetostrictive properties^5^,^6^,^7^. LSMO can be coherently grown on commercially available perovskite substrates, such as NdGaO_3_ and DyScO_3_, and depending upon the substrate, the induced epitaxial strain can be compressive or tensile. In a recent work, it was demonstrated that La_0.7_Sr_0.3_MnO_3_ deposited on DyScO_3_ exhibits anisotropic transport properties due to a substrate-induced tensile strain of 1.9%^8^. In addition, many studies have been carried out to investigate strain effects in either bulk or thin-film manganites to elucidate the fundamental coupling mechanisms and to explore design rules for devices with improved performance^7^,^9^,^10^.

Oxygen vacancy defects are believed to play an important role in giving rise to the unique properties of perovskite manganese oxides. For example, a two-dimensional electron gas has recently been found at the interface between SrTiO_3_ and LaAlO_3_ and the origin of the charge in heterostructures comprised of these two materials is attributed to the oxygen vacancies^11^,^12^. Picozzi *et al.* reported that the oxygen vacancies cause prominent changes in the electronic and magnetic structures of La_0.66_Sr_0.33_MnO_3_^13^. It was also found that the valence band features shift toward higher binding energies and the degree of covalency of Mn bonding increases. From the crystallographic perspective, the MnO_6_ octahedron is an important feature of the perovskite manganeseoxides, and the existence of an oxygen vacancy in an octahedron leads to a change in its structure via correlated deformation and rotation of neighboring octahedra. Such structural changes of MnO_6_ octahedron may, in turn, play an important role in influencing the electronic and magnetic properties. Therefore, the issue of the influence of oxygen vacancies of LSMO system should be paid more attention.

In this study, we have carried out the density functional theory (DFT)-based first principles calculations by using the generalized gradient approximation (GGA)+U formalism to investigate the oxygen vacancy induced changes in the structural, electronic and magnetic properties LSMO system. In addition, the formation energy of an oxygen vacancy with different charge state and the stability of different charge state are also studied.

## Results

### Structural comparison between vacancy-containing and pristine LSMO system

**Figure 1:**
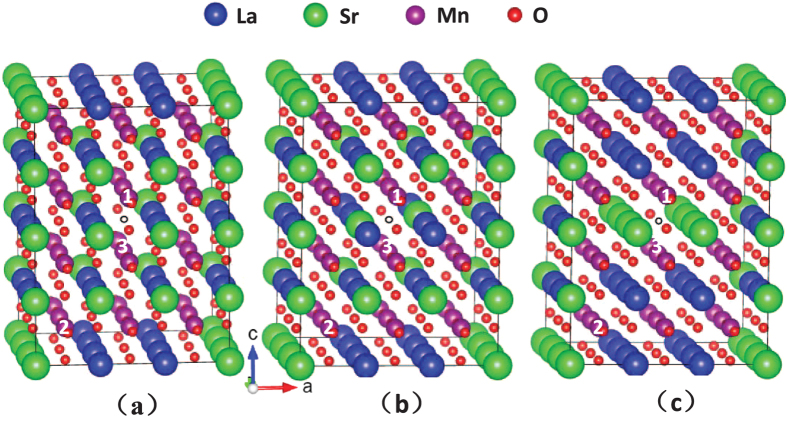
Three configurations of 3 × 3 × 4 supercell for La_1−*x*_A_*x*_MnO_3_ (*x* = 1/3) containing one oxygen vacancy at the center denoted by a hollow circle. The number of Sr atoms (green spheres) as first-nearest neighbors of the vacancy in the ab-plane is (**a**) zero (**b**) two and (**c**) four. The blue, pink and red spheres correspond to La, Mn and O atoms, respectively. Mn Atoms labeled as 1 (Mn_1_) and 2 (Mn_2_) reside in the first-nearest neighboring and the third-nearest neighboring MnO_6_ octahedron of the acancy, respectively, while 3 (Mn_3_) is a first-nearest neighbor of the vacancy.

**Figure 2:**
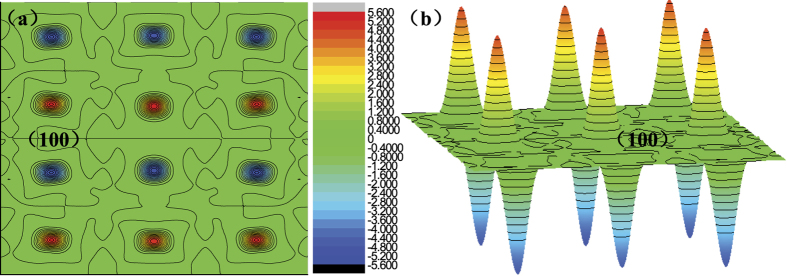
The calculated local magnetic alignments for La_1−*x*_Sr_*x*_MnO_3_ (*x* = 1/3). (**a**) spin density along (100) plane where the circle regions denote the Mn site. (**b**) an illustration of alignment of Mn moment in (100) plane where the peaks outside the plane denote the difference of electron density between the two spin directions. The maximum value and minimum value is 5.6 e/Å^3^ and −5.6 e/Å^3^, respectively, and the contour step size is 0.4 e/Å^3^.

First, we have carried out geometry optimization on the three supercell configurations shown in Fig. 1(a–c) using the GGA+U formalism, the calculated energy difference between cases (a) and (b) in Fig. 1 is −0.26 eV, and the energy difference between cases (a) and (c) in Fig. 1 is −0.35 eV. Therefore, the structure shown in Fig. 1(a) with no Sr atoms present at the first-nearest neighbor sites of vacancy in the ab-plane, is the most stable among the three cases. Then, we carried out geometry optimization calculations on this structural model using the GGA as well as the GGA+U method, in which the Hubbard U parameter has been determined by linear response theory (LRT) to be 5.9 eV. Another issue that should be predetermined is the magnetic ordering. We calculated the total energy of two kinds of magnetic ordering, i.e. ferromagnetic and A-type antiferromagnetic ordering, to determine which is more stable. Furthermore, in addition to using U = 5.9 eV, we also used U = 2.0 eV and U = 0 eV for that magnetic ordering calculation in order to check the magnetic stability with the variation of U value. The results show that the energy of A-type antiferromagnetic ordering is 0.8 eV lower than that of ferromagnetic ordering for U = 5.9 eV. In addition, the energy of A-type antiferromagnetic ordering is also lower than the ferromagnetic ordering for U = 2.0 eV and U = 0 eV, and that energy difference is 1.2 eV and 0.9 eV for U = 2.0 eV and U = 0 eV, respectively. Therefore, the A-type antiferromagnetic ordering is more stable than the ferromagnetic ordering and the stability is maintained with changing the U value, and all the following calculations are based on A-type antiferromagnetic ordering. To see the alignment of Mn moment clearly, we show the calculated spin density along (100) plane of 3 × 3 × 4 supercell with A-type magnetic ordering in Fig. 2. Here, the spin density is defined as the density difference between spin up and spin down electrons, and the positive value suggests that the electrons numbers with up spin are more than that with down spin per unit volume, and vice versa. From Fig. 2(a), we see that the Mn moment align parallel in the ab-plane while oppositely for the two neighboring atomic layers along c direction. For more straightforward understanding, we can refer to the Fig. 2(b) where the red and blue peaks outside the (100) plane denote the difference of electron density for Mn atoms between the two spin directions.

**Figure 3:**
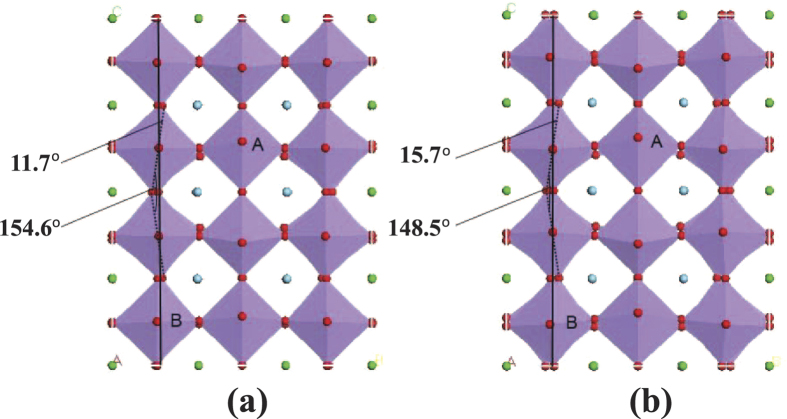
The profile in the bc-plane of the La_1−*x*_Sr_*x*_MnO_3_ (*x* = 1/3) supercell obtained by structural relaxation with the (**a**) GGA method and (**b**) GGA+U method. Rotation of MnO_6_ octahedra (with Mn atoms not visible) to various degrees is clearly evident. The upper and lower arrows denote the maximum angle of rotation with respect to the c-axis and minimum Mn-O-Mn bond angle, respectively. The solid black line is added as visual aid to indicate the vertical direction. The MnO_6_ octahedra labeled A and B respectively correspond to the first-nearest neighbor and the third-nearest neighbor of the oxygen vacancy.

The profiles in the bc-plane of the relaxed supercell are shown in Fig. 3. It can be seen that all the MnO_6_ octahedra show various degree of rotation with respect to the c-axis and the ab-plane. The GGA+U method results in a larger angle of rotation than the GGA method. For example, the maximum angles of rotation with respect to the c-axis are 11.7° and 15.7° for GGA and GGA+U method, respectively. The Mn-O-Mn bond angles get reduced due to such rotation of the octahedra, to as low as 154.6° with the GGA method and 148.5° for the GGA+U method. Furthermore, the optimized geometries exhibit not only the rotation of the MnO_6_ octahedra, but also their deformation. Various structural parameters characterizing the relaxed systems are listed in Table 1. We first focus on the atoms surrounding the oxygen vacancy. The oxygen vacancy has two Mn atoms as the first-nearest neighbors, four La atoms as the second-nearest neighbors, and eight O atoms as the third-nearest neighbors. Compared with the pristine system, the two Mn atoms of the vacancy system move closer to each other by about 0.21 Å and 0.24 Å for GGA and GGA+U, respectively. Like the Mn atoms, the second-nearest neighboring oxygen atoms has also tendency of moving closer induced by oxygen vacancy, and the distance change is about 0.5 Å and 0.6 Å for GGA and GGA+U, respectively. However, the third-nearest neighboring La atoms move away from each other, and the distance change is about 0.16 Å both for GGA and GGA+U. Actually, the change of atom-atom distance surrounding oxygen vacancy should be ascribed to the rotation and deformation of MnO6 octahedra surrounding oxygen vacancy. Moreover, the more reduction of Mn–Mn and O–O distance for GGA+U than GGA results from the more stronger structural change of MnO_6_ octahedra than that of GGA. Here, the oxygen-vacancy induced reduction of Mn–Mn distance is similar to the case of SrTiO_3_ where the Ti–Ti distance is found to contract after the relaxation of vacancy-containing system using the HSE method^11^. Furthermore, the degree of deformation for MnO_6_ octahedra is different along different directions. In Table 1, we have listed the structural parameters of the octahedron A which is the first-nearest neighbor of the oxygen vacancy in bc-plane (see Fig. 3). The deformation causes the O–O distance along a, band c axes to generally increase slightly after the relaxation of vacancy-containing system as compared to the pristine system, except for its value along b-axis given by for GGA method which remains unchanged. In summary, the GGA+U method results in more prominent changes in the structure than that of GGA method. In the next section, we examine the possible influence of such structural changes on the electronic and magnetic properties of the system studied.

### The change of Mn-3d orbital electronic structure induced by introducing Hubbard U

**Figure 4:**
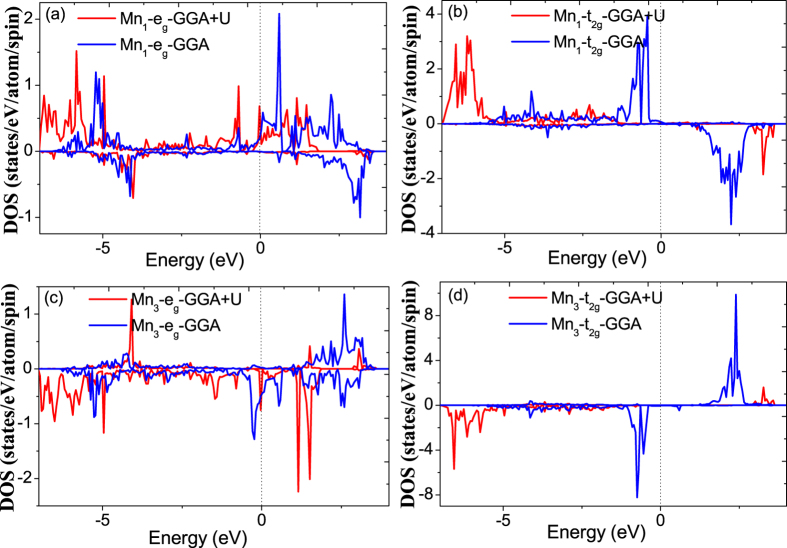
The calculated 3d orbital DOS for the Mn atoms located in two typical sites. Mn_1_ corresponds to the atom located in octahedron A; Mn_3_ corresponds to the atom located in the first-nearest neighboring site of the oxygen vacancy. (**a**) e_g_ DOS of Mn_1_ by GGA and GGA+U. (**b**) t_2g_ DOS of Mn_1_ by GGA and GGA+U. (**c**) e_g_ DOS of Mn_3_ by GGA and GGA+U. (**d**) t_2g_ DOS of Mn_3_ by GGA and GGA+U.

Like the resulting prominent structural difference between GGA and GGA+U structural relaxation, the electronic structure especially for Mn atoms should be also different between the two methods. In order to explore the difference of Mn-3d orbital electronic structure between GGA and GGA+U method, we show the calculated density of states (DOS) of e_g_ and t_2g_ orbitals for Mn_1_ (locates in MnO_6_ octahedron A) and Mn_3_ (locates in the first-nearest neighboring site of oxygen vacancy) in Fig. 4. In addition, we remark that the Mn_2_ denotes the atom located in MnO_6_ octahedron B and the definition for Mn_1_, Mn_2_ and Mn_3_ is also coherent in all the following discussions. On the whole, the introduction of Hubbard U lead to a prominent change of Mn 3d electronic structure for both Mn_1_ and Mn_3_. The GGA+U DOS of e_g_ orbital does not exhibit any marked changes in the features as compared to the GGA DOS, especially in the low energy region. However, for t_2g_ orbital the GGA+U DOS results in that the localized splitting peaks of majority and minority spin peaks lie far from the Fermi level due to the strong Coulomb repulsion. For instance, the majority spin peak for GGA+U and GGA DOS is located in −6.0 eV and −1.0 eV, respectively, resulting in a 5.0 eV shift. This feature indicates the effect of Hubbard U on Mn 3d localized orbital. Actually, these features of electronic structure induced by introducing Hubbard U is the basis of correctly deal with the whole LSMO system. Because we can safely assume that the GGA+U method can give the correct description for LSMO system, then in the following discussions we only concentrate our analysis on the GGA+U results.

### The relative stability of oxygen-vacancy-containing LSMO system with different charge state

**Figure 5:**
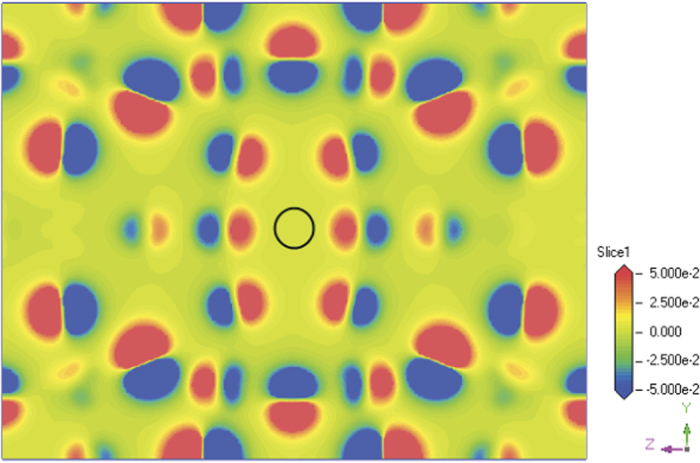
The calculated difference of electron density between charge state q = −2 and q = 0. The black circle denotes the position of the oxygen vacancy.

We consider the charge state q = 1, −2, +1 and +2 for the oxygen-vacancy-containing system, and the calculated total energy for different charge state as well as the neutral state q = 0 is listed in Table 2. It can be seen that the charge state q = −2 has the lowest formation energy indicating it is most stable among the different charge states. Therefore, the oxygen vacancy here is a hole-type defect, that is, it has a tendency of attracting electrons. In order to look close into this feature, we have calculated the difference of electron density between charge state q = −2 and neutral state q = 0, and the result is shown in Fig. 5 which clearly shows the electron density distribution of the net −2 charge. We can see that the oxygen vacancy region is surrounded by six red regions which represent the electron density concentration, indicating that it lead to a local negative charge concentration. This feature of negative charge of oxygen vacancy should be the origin of structural contraction surrounding the oxygen vacancy as mentioned above. Moreover, this charge character of oxygen vacancy may have an important influence on conduction of this material which needs to be confirmed by experiment.

### The comparison of DOS for Mn and O between the oxygen-vacancy-containing and pristine system

**Figure 6:**
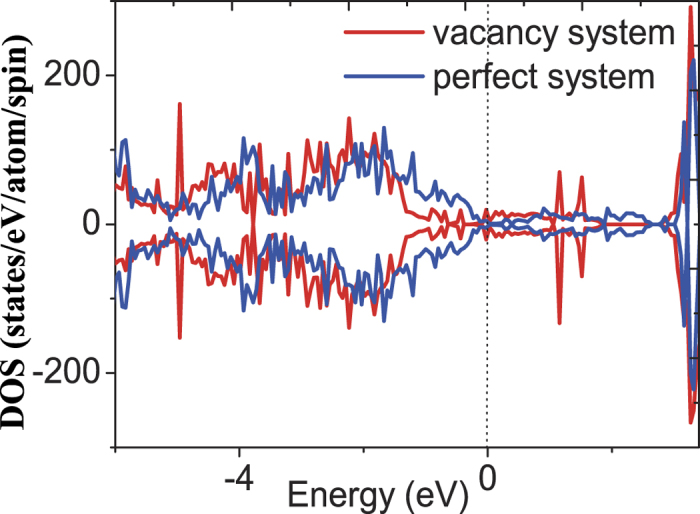
The calculated total DOS of La_1−*x*_Sr_*x*_MnO_3_ (*x* = 1/3) compound for oxygen-vacancy-containing and pristine systems. The vertical dotted line denotes the Fermi level.

The calculated total DOS of La_1−*x*_Sr_*x*_MnO_3_ (*x* = 1/3) for oxygen-vacancy-containing and pristine systems are depicted in Fig. 6. The antiferromagnetic character of this system becomes clearly evident from the symmetric spin-up and spin-down DOS for both vacancy and pristine system. In addition, they both exhibit a typical metallic nature since the Fermi level passes through the band. Hence we see that the La_1−*x*_Sr_*x*_MnO_3_ (*x* = 1/3) system has changed its nature from ferromagnetic half-metal for bulk material to antiferromagnetic metal for tensile strained film induced by substrate^8^. The DOS of vacancy case exhibits more or less states rearrangement compared to that of pristine, especially for the region around the Fermi level. The states decreased in the range from −1.7 eV to −0.1 eV and increased in the range from −0.1 eV to 0.7 eV. Another clear change can be found that three peaks at −5.0 eV, 1.0 eV and 1.5 eV, respectively, appear in vacancy system DOS.

**Figure 7:**
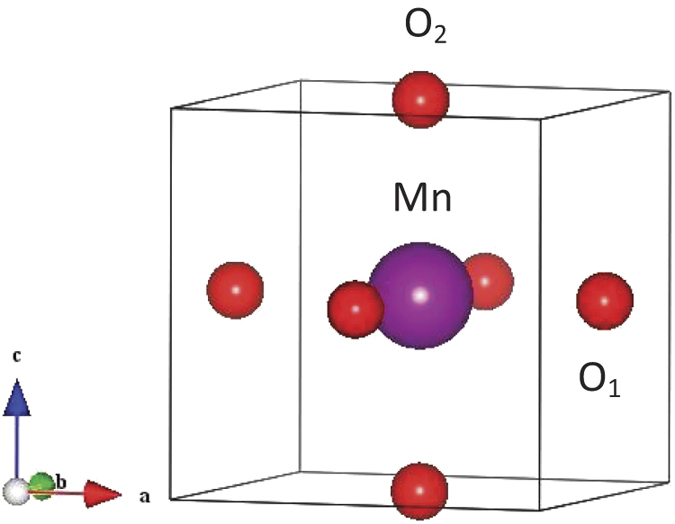
The structural illustration for one MnO_6_ octahedron where O_1_ bonds to the Mn atom in a direction and O_2_ bonds to the Mn atom in c direction.

**Figure 8:**
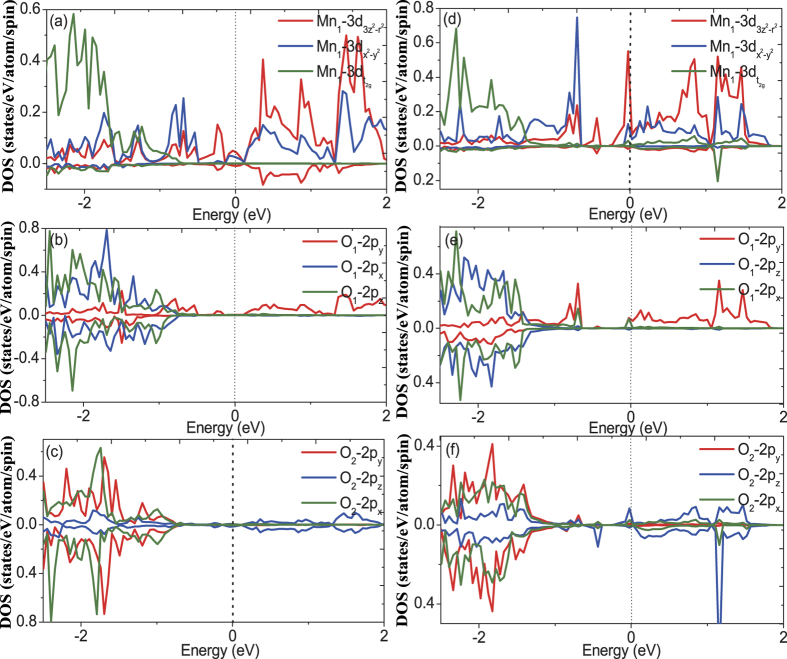
The calculated orbital DOS for Mn and O of MnO_6_ octahedron A for vacancy and pristine systems. (**a**) Mn 3d orbital DOS of pristine system. (**b**) O_1_ 2p orbital DOS of pristine system. (**c**) O_2_ 2p orbital DOS of pristine system. (**d**) Mn 3d orbital DOS of vacancy system. (**e**) O_1_ 2p orbital DOS of vacancy system. (**f**) O_2_ 2p orbital DOS of vacancy system.

For the partial DOS calculations, we choose several typical sites for Mn and oxygen atoms. First, octahedron A and B as shown in Fig. 3 corresponds to the first-nearest neighbor and the third-nearest neighbor of the oxygen vacancy, respectively. Second, the O_1_ and O_2_ denote the oxygen atom bonding to Mn atom in the ab-plane and along c axis for one MnO_6_ octahedra, respectively, shown in Fig. 7. In Fig. 8 we show the orbital DOS for Mn and oxygen atoms of octahedron A for the pristine system and oxygen-vacancy-containing system. It is clear that for the pristine system, the two e_g_ DOS of Mn, i.e. 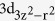 and 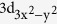, occupy the high energy regime whereas the t_2g_ DOS of Mn occupy the low energy regime, consist with the general character of electronic structure for LSMO system^14^. The 2p_y_ DOS of O_1_ and 2p_z_ DOS of O_2_ dominate the same high energy regime with that of Mn, indicating the hybridization of Mn−O_1_ in ab-plane and the hybridization of Mn−O_2_ along c axis, respectively. In addition, for vacancy system, the Mn t_2g_ DOS appears in the high energy regime and exhibits a ~0.2 eV peak in the minority spin states, and this large exchange splitting of t_2g_ states should be attributed to the oxygen vacancy induced rotation and deformation of MnO_6_ octahedra. This prominent difference of Mn DOS between the vacancy system and pristine system may lead to corresponding change of magnetic properties which will be seen in the following discussions.

**Figure 9:**
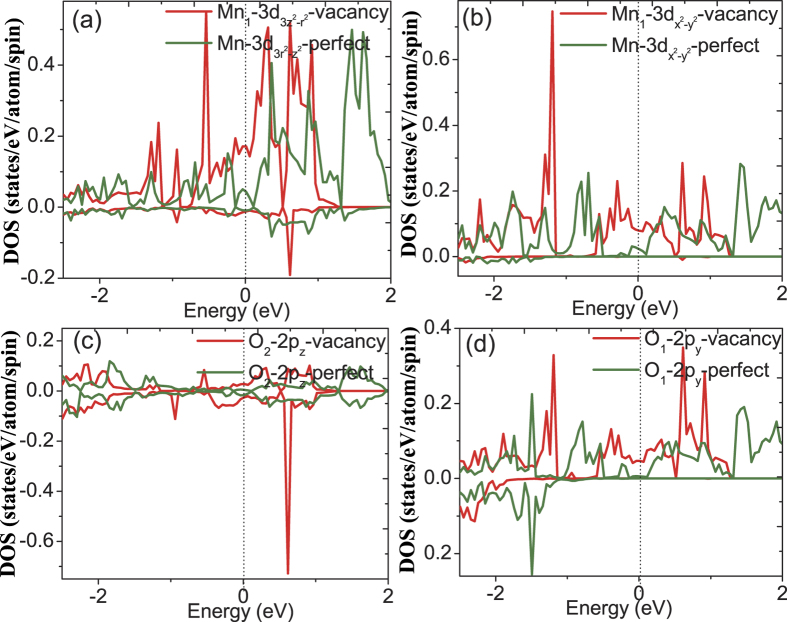
The calculated orbital DOS for (**a**) Mn_1_−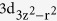, (**b**) Mn_1_−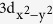, (**c**) O_1_−2p_z_ and (**d**) O_1_−2p_x_ of octahedron A for pristine system and vacancy system.

**Figure 10:**
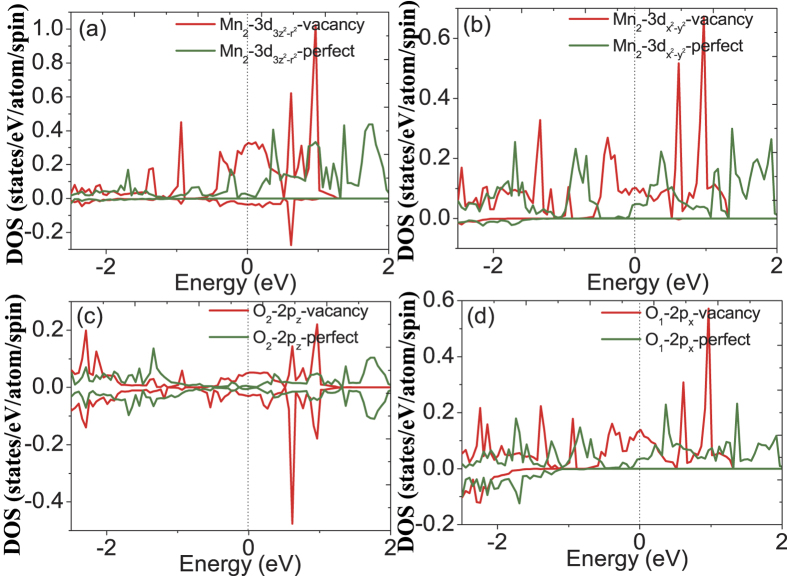
The calculated orbital DOS for (**a**) Mn_1_−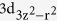, (**a**) Mn_1_−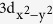, (**c**) O_1_−2p_z_ and (**d**) O_1_−2p_x_ of octahedron B for pristine system and vacancy system.

In order to further explore the changes in the electronic structure induced by the oxygen vacancy, we have also plotted the orbital DOS of Mn, O_1_ and O_2_ belonging to octahedral A and B, respectively. All of these figures also show the corresponding results for the pristine supercell for easier comparison. In the results for the octahedron A shown in Fig. 9, O_1_ and O_2_ states are observed to hybridize with Mn atom in b and c directions, respectively. It can be seen that the Mn−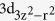 and Mn−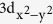 DOS at the Fermi level have increased after relaxation of the oxygen-vacancy-containing structure, and the O_1_−2p_y_ and O_2_−2p_z_ DOS also exhibit slight enhancement. This indicates that the hybridization between Mn-3d orbital and O-2p orbital has been strengthened along both the c and b axes. Similar nature of the DOS has been also found for octahedron B as shown in Fig. 10. The Mn−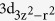 and Mn−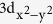 DOS at the Fermi level for the vacancy system are larger than that of pristine system, and similarly trend is found for O_1_−2p_x_ and O_2_−2p_z_ DOS. This again indicates that the hybridization between Mn-3d and O-2p orbital has been strengthened along both the c and a axes. This change in the orbital DOS can be clearly ascribed to the structural deformation induced by the oxygen vacancy in the system.

**Figure 11:**
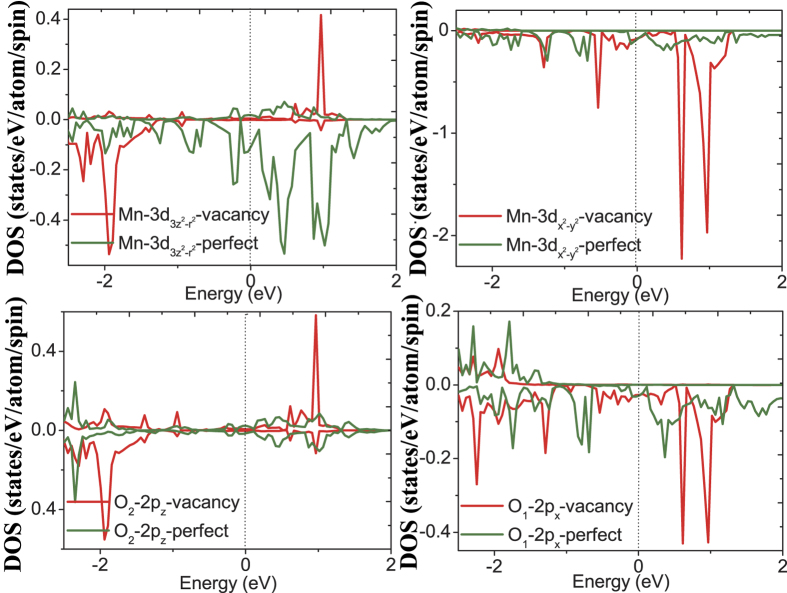
The calculated orbital DOS for the first-nearest neighboring Mn atoms surrounding the oxygen vacancy.

**Figure 12:**
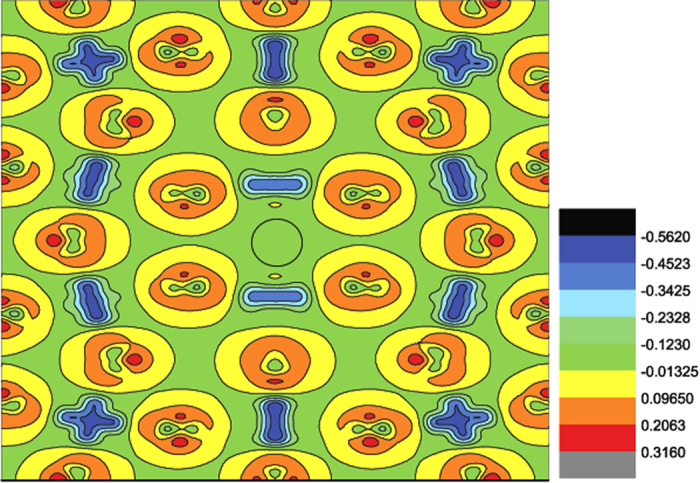
The calculated bonding electron density in bc plane where the oxygen vacancy resides in the black circle denotes the position of oxygen vacancy. The maximum value and minimum value is 0.3160 e/Å^3^ and −0.5620 e/Å^3^, respectively, and the contour step size is 0.1097 e/Å^3^.

The calculated orbital DOS for the first-nearest neighboring Mn atom surrounding the oxygen vacancy, i.e. Mn_3_ and corresponding O_1_ and O_2_ which bond to Mn in a and c direction, respectively, is shown in Fig. 11. For the 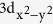 orbital, the vacancy and the pristine system have almost the same magnitude of DOS at the Fermi level, however, for the 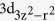 orbital, the vacancy system DOS are clearly smaller than the pristine system DOS at the Fermi level, indicating that the hybridization along z direction between Mn-3d and O-2p orbital has weakened. This decreased hybridization of z direction appears to originate from its losing bond to Mn atoms in the z direction due to introduction of oxygen vacancy. In order to explore the change of the Mn-O interaction, we plot the bonding electron density in bc-plane including the oxygen vacancy in Fig. 12. The bonding electron density is defined as the difference between the total electron density in the solid and the superposition of neutral atomic electron densities placed at the same atomic sites. Therefore, it represents the net electron redistribution as atoms are brought together to form the crystal. The positive and negative values represent gaining and losing electrons, respectively. We can see that the electrons accumulate around the O atoms and deflect around the Mn atoms, basically showing an ionic characteristics of the Mn-O bond. In addition, a clear feature can be found that the two first-nearest neighboring Mn atoms surrounding the oxygen vacancy have different contour profiles with that in other positions, and the distribution of electron density along c axis has decreased and that along b axis has increased. This change of electron density distribution for the first-nearest neighboring Mn atoms indicates a remarkable change of Mn-O interaction, and the Mn-O interaction has weakened in c axis direction and strengthened in ab-plane due to the introduction of oxygen vacancy, consistent with the above analysis of corresponding orbital DOS.

### The comparison of magnetic properties between the oxygen-vacancy-containing and pristine system

In order to explore the change of magnetism between oxygen-vacancy-containing and pristine system, we show the calculated magnetic moments, total valence electron number (VEN) and valence electron configuration of Mn_1_ and Mn_3_ atoms in Table 3. For Mn_1_ and Mn_3_, the enhanced moment is 0.141 μ_B_ and 0.144 μ_B_, respectively. We have not shown the moments of La, Sr and O atoms because their contribution to the total magnetism can be negligible. It can be seen that the Mn moments in the two typical sites of the vacancy system are larger than that of pristine system. The enhancement of Mn magnetic moments should be attributed to the change of complex interaction between Mn atoms and other atoms especially O atoms induced by octahedral rotation and deformation. To understand the electronic origin for the change of magnetism, we also calculated the valence electron configuration using Bader charge analysis for both vacancy and pristine system. For Mn_1_ atom, the vacancy and pristine system has almost the same value of VEN, ~5.023. However, for Mn_3_ atom, the VEN of vacancy system has prominent enhancement, 0.134, compared to that of pristine system. This enhanced total VEN for vacancy system can be explained by the reduction of losing electrons due to absence of one Mn-O bond caused by the oxygen vacancy. From the valence electron configuration of Mn_1_ atom, we can see that there are 0.07 electrons transfer from 3p to 3d orbital, and this transfer of electrons lead to the enhancement of magnetic moment because the five 3d orbital tend to accommodate five electrons with parallel spin moment according to the Hund’s Rule. However, unlike Mn_1_ atom, the enhanced 0.06 electrons of Mn_3_ atom mainly come from the total VEN while not 3p orbital. On the whole, we see a prominent change of magnetism for Mn atoms, and this change result from the structure induced change of bonding properties and finally the electrons transfer caused by oxygen vacancy.

### The orbital and charge ordering caused by the tensile strain for pristine and oxygen-vacancy-containing system

**Figure 13:**
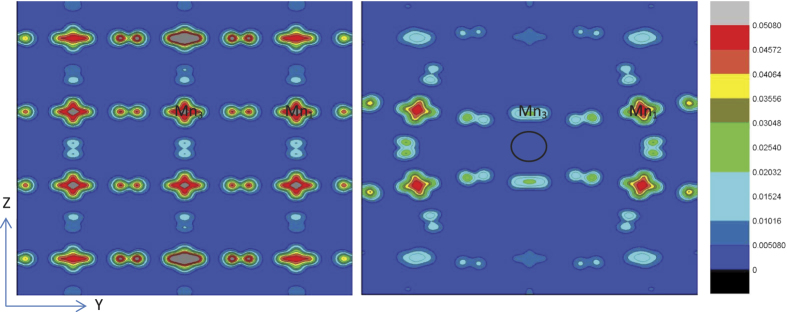
Valence electron charge density contours plotted on the (100) plane in the energy range E_F_ − 0.3 eV to E_F_ to indicate the orbital ordering. The left and right panel denotes the pristine system and oxygen-vacancy-containing system, respectively. The black circle refers to the position of oxygen vacancy. The value of charge density is from zero to 0.0508 e/Å^3^ and the step size is 0.00508 e/Å^3^.

It is well known that the substrate distorts the MnO_6_ octahedra and splits the degenerate Mn e_g_ states into x^2^ −y^2^ and 3z^2^ −r^2^ state, and compressive strain makes 3z^2^ −r^2^ lower in energy and tensile strain makes x^2^ −y^2^ lower in energy^15^. Therefore, the valence electrons would prefer to occupy the corresponding lower orbital with strain. In our case of La_1−*x*_Sr_*x*_MnO_3_ (*x* = 1/3) system with a 1.9% tensile strain distorted by the substrate DyScO_3_, The preferable occupation for e_g_ orbital should be x^2^ −y^2^. To see this orbital occupation character, we calculated the valence electrons charge density in the vicinity of the Fermi level, shown in Fig. 13 providing a visualization of the above analysis. First, we focus on the pristine system shown in the left panel. Since all the atoms are located in the periodical position, the arrangement of charge density of Mn and O atoms is very orderly. It is clear that all the Mn atoms show preferable occupation of the x^2^ −y^2^ orbital in agreement with the above analysis. In addition, we note that the O atoms show the 2p_y_ orbital character in the y direction and 2p_z_ orbital character in the z direction. Second, we concentrate on the case of oxygen-vacancy-containing system shown in the right panel. Unlike the pristine system, the charge density of Mn and O atoms for oxygen-vacancy-containing system show different degrees of rotation due to the structural rotation of MnO_6_ octahedra. We can find a clear feature that the Mn atoms in different sites show different orbital occupation character. For comparison, we label the site of Mn_1_ and Mn_3_, and Mn_1_ shows mixture of occupation for x^2^ − y^2^ and 3z^2^ − r^2^ orbital whereas Mn_3_ shows almost complete occupation of x^2^ − y^2^ orbital with almost no occupation character of 3z^2^ − r^2^ orbital. This reason should be due to the weakening of hybridization with 2p states of O atoms in z direction because of the existence of oxygen vacancy. On the whole, we see that the Mn atoms of oxygen-vacancy-containing system show a different orbital occupation character with that of pristine system though they are both under the tensile strain, and the Mn1 atoms does not show a preferable x^2^ − y^2^ occupation but a mixture of x^2^ − y^2^ and 3z^2^ − r^2^ occupation and Mn_3_ shows a much stronger x^2^ − y^2^ occupation with almost no 3z^2^ − r^2^ occupation due to losing a bond with O atoms.

In summary, first principles calculations using GGA+U formalism have been carried out for oxygen vacancy-containing La_1−*x*_Sr_*x*_MnO_3_ (*x* = 1/3) system, focusing on the oxygen vacancy induced changes in structural, electronic and magnetic properties compared with the corresponding pristine LSMO system. The structural optimization of vacancy system shows that there are prominent rotation and deformation of MnO_6_ octahedra. In addition, the first-nearest neighboring Mn atoms and the third-nearest neighboring O atoms surrounding the oxygen vacancy move closer to each other than that of pristine system, and the second-nearest neighboring La atoms move away from each other than that of pristine system. The GGA+U results show a more remarkable structural change than the GGA results. By the calculation of orbital DOS for Mn and O in three typical sites, we found that for the first-nearest neighboring Mn atoms surrounding the oxygen vacancy, the hybridization between Mn-3d and O-2p has been strengthened in ab-plane while weakened along c axis direction because the Mn atoms has lost a bonding to oxygen in c direction. For the other two sites, the hybridization between Mn-3d and O-2p has become stronger both for ab-plane and c axis direction due to the strong structural change of MnO_6_ octahedra. The calculations of magnetic moment for Mn_1_ and Mn_3_, locate in the first nearest-neighboring site and the first nearest-neighboring MnO_6_ octahedra of oxygen vacancy, respectively, show that the Mn moments for the two typical sites of vacancy system are all larger than that of pristine system. This enhanced Mn moment result from the increased valence electrons of 3d orbital induced by the structural change due to oxygen vacancy. By calculating the formation energy of oxygen vacancy with different charge states, it is found that the oxygen vacancy with q = −2 is most stable indicating its character of negative charge center. The e_g_ orbital occupation character is compared between the pristine system and oxygen-vacancy-containing system by calculating the valence electrons charge density, and the results show that the Mn atoms in pristine system show preferable x^2^−y^2^ occupation due to the tensile strain induced by DyScO_3_ substrate in agreement with the well known view of point, however, for the vacancy-containing system, the Mn atoms in different sites show different orbital occupation character due to the strong rotation of MnO_6_ octahedra and existence of oxygen vacancy. The Mn_1_ atoms show a mixture of x^2^−y^2^ and 3z^2^−r^2^ occupation and Mn_3_ shows a much stronger x^2^−y^2^ occupation with almost no 3z^2^−r^2^ occupation due to losing a bond with O atoms.

## Methods

In our study, a perovskite unit cell with formula La_1−*x*_Sr_*x*_MnO_3_ (*x* = 1/3) is used, and the lattice parameters are a = 3.952 Å, b = 3.947 Å and c = 3.788 Å, chosen according to experimental values reported for LSMO film samples grown on DyScO_3_, which is in a 1.9% in-plane tensile strain with respect to the unstrained lattice parameter 3.876 Å^8^. In order to avoid the strong interactions between the defects, we choose a sufficiently large 3 × 3 × 4 supercell for the perovskite unit cell La_1−*x*_Sr_*x*_MnO_3_ (*x* = 1/3), which contains 180 atoms. An oxygen vacancy is introduced by removing one O atom located at the center of the supercell. In fact, there are several possible occupation sites for vacancy. In other words, the surrounding atoms neighboring the vacancy have several possible cases. Therefore, we calculated the three possible structural models, i.e. the number of Sr atoms as first-nearest neighbors of the vacancy in the ab-plane is zero two and four, which are shown in Fig. 1(a–c). The number of Sr atoms located at the first-nearest neighbor sites of the vacancy defect in the ab-plane is zero in Fig. 1(a), two in Fig. 1(b) and four in Fig. 1(c). The most energetically favorable configuration is then determined via the calculation of the total energy, and used for all the subsequent calculations. In the calculations of structural optimization, the lattice parameters and symmetry of supercell are fixed and the atomic positions are relaxed until the forces reach the required accuracy. As for the magnetic ordering, it should be noted that the epitaxial La_0.7_Sr_0.3 _MnO_3_ film deposited on DyScO_3_ substrate reported by ref. [8] has been shown to exhibit an A-type antiferromagnetic ground state due to the tensile strain induced by substrate, consistent with the theoretical phase diagram calculated by Fang *et al.*^16^. The so-called A-type antiferromagnetic ordering means that the magnetic moments of all the Mn atoms in one layer parallel to the ab-plane have an uniform in-plane direction, while the moment direction is opposite for the two neighboring atomic layers. Although the Sr-concentration 1/3 in our model system deviates slightly from 0.3, it is still in the A-type antiferromagnetic regime according the predicted phase diagram. In addition, the experimental investigations have shown that La_1−*x*_Sr_*x*_MnO_3_ (*x* = 1/3) belongs to antiferromagnetic metal^17^,^18^. Nonetheless, we also carried out total energy calculation for A-type antiferromagnetic and ferromagnetic ordering to confirm the magnetic ordering.

It is well known that first principles calculation based on DFT for the strongly correlated systems (e.g., the LSMO system) should use the formalism going beyond the local density approximation (LDA) and GGA methods. Such formalism include GGA (LDA)+U, GW and hybrid functionals, etc, which yield band gaps in better agreement with the experimental values. The GGA+U is an effective approach to treat systems containing transition metals and further it is computationally more efficient than the GW and hybrid functionals approaches. Our first principles calculations are performed by using the plane-wave pseudopotential code Vienna ab initio simulation package (vasp)^19^,^20^. The core–electron interactions are modeled by the projector-augmented wave potentials^21^and the Perdew-Burke-Ernzerh of-correlation functional is adopted^22^,^23^. As energy functionals, the standard formalisms of GGA and GGA+U (U is the on-site Coulombic repulsion) are used, and we only consider U value for Mn d electrons calculated by the linear response theory. A detailed description of calculation of U is provided in the following section. The plane-wave cutoff energy was chosen as 400 eV, and the Brillouin zone was sampled by using 3 × 3 × 1 Gamma centered Monkhorst-Pack k-points grid in performing energy minimization and 5 × 5 × 3 Gamma centered Monkhorst-Pack k-points grid in the subsequent electronic properties calculations. The convergence criterion for energy and force was set to 10^−5 ^eV/unit cell and 0.01 eV/Å, respectively.

**Figure 14:**
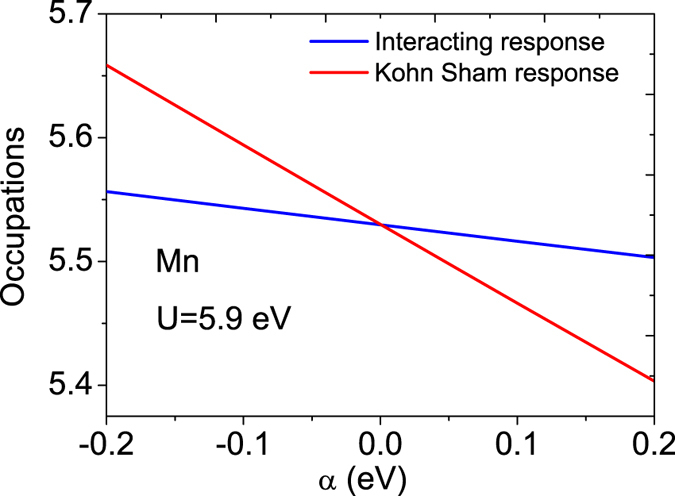
Linear response of d orbital occupations to the change of potential shift α. The red and blue lines represent the Kohn-Sham and the noninteracting inverse density response functions, respectively.

In the GGA+U formalism, the value of the on-site Coulombic repulsion parameter U depends upon which compound the transition metal is a constituent of. For example, U = 3 eV was employed in modeling of the magnetic properties of La_0.625_Sr_0.375 _MnO_3_ under high pressure^24^, whereas for LaMnO_3_ the value of U was chosen to be 4 eV^25^. Previous studies based on first principles calculations using GGA (LDA)+U approach for perovskites containing Mn provide no consensus on the appropriate value of U. The Hubbard parameter U can, in fact, be calculated by the LRT proposed by Cococcioni and Gironcoli *et al.*^26^ who have demonstrated the accuracy of this method by computing structural and electronic properties of various systems including transition metals, rare-earth correlated metals and transition metal monoxides. We have thus calculated the effective on site Coulomb repulsion parameter U_eff_ for La_1−*x*_Sr_*x*_MnO_3_ (*x* = 1/3) by using the LRT that is internally consistent with the chosen definition for the occupation matrix of the relevant localized orbitals^26^,^27^. According to such LRT, The effective interaction parameter U_eff_ associated to site *I* can be written as


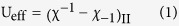


where 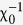 and 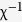 are the interacting and non interacting Kohn-Sham density response functions of the system, respectively. They can be calculated by 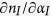 and 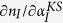, respectively, where *n*_*I*_ is the atomic orbital occupations for the atom *I*, and *α*_*I*_ and 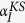 denote the interacting and non interacting Kohn-Sham localized perturbation potential, respectively. Using this method, we have calculated the response function of Mn and the resulting plot of d orbital occupation versus α is shown in Fig. 14. By changing the rigid potential shifts α, we obtain the bare and self-consistent occupation regression response functions. The interacting 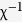 and the Kohn−Sham 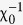 inverse matrices are the slopes of bare and self-consistent regression response functions, respectively. The resulting value of the Hubbard parameter U_eff_ for Mn atom calculated from e.q. (1) is 5.9 eV. The reliability of this calculated value of U_eff_ can be further exemplified by noting that it is close to the value employed in our previous first principles calculations (U_eff_ = 6.0 _e_V) which yielded electronic structure results consistent with the X-ray absorption spectroscopic data^8^.

The relative stability of oxygen vacancy with various charge states is determined by the formation energy. The formation energy of an oxygen vacancy in La_1−*x*_Sr_*x*_MnO_3_ (*x* = 1/3) can be calculated from the total energy of the supercell using the Zhang-Northrup formalism^28^. According to this standard formalism, the defect formation energy for a metallic compound with charge state q is dependent on atomic and electronic chemical potential and is given by


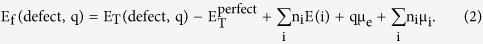


where E_f_ (defect, q) is the formation energy of a defect in charge state q, i.e. the number of electrons transferred from the supercell to the reservoirs in forming the defect. E_T_ (defect, q) and 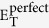 denote the total energy of the compound containing a defect in charge state q, and the pristine compound in the same supercell size, respectively. n_i_ represent the number of atoms removed from the compound, to form the defect while E(i) denotes the total energy of the ith atom. μe is the chemical potential of electron, i.e. the Fermi level, and μ_i_ is the chemical potential of ith atom referenced to elemental solid/gas with energy E(i). Generally, two energy corrections should be considered because of the interaction between the charged defect and the compensating background and its periodic images. One is the correction of valence band maximum considering that the value of valance band maximum is different for supercell with and without a defect owing to the defect-induced distortion of the band structure near the band edges^29^,^30^,^31^,^32^,^33^, and here we use this correction for Fermi level μ_e_ of which the value is referenced to valence band maximum. Such correction can be done as this method that we assume the potentials in the pristine system are similar to those far from a defect in defective system. Then, the average potential of the plane farthest from the defect in the defective system 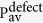 and the average potential of the corresponding plane in the pristine system 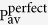 are determined, and the difference in the averaged potentials between the defective and pristine system can be used to correct μ_e_ as follows


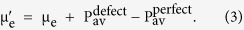


Another is the Makov-Payne (M–P) correction, q^2^α/2εL, where ε is the static dielectric constant, L is the linear dimension of the supercell, and α is the Madelung constant^34^, and it should be considered in our case. This M-P correction term describes the electrostatic energy of the point charge array in a uniform background in the presence of screening medium described by the static dielectric constant^31^. For the system studied in this work, the formation energies of oxygen vacancies at different charge states in La_1−*x*_Sr_*x*_MnO_3_ (*x* = 1/3) can be written as


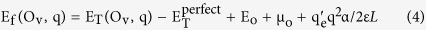


where E_f_ (O_V_, q) is the formation energy of oxygen vacancy in charge state q, E_T_ (O_V_, q) and 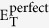 are the total energies of the supercells with and without oxygen vacancy and E(O) denotes the energy of an oxygen atom taken to be equal to half of that of an oxygen molecule E(O_2_)^35^. μ_O_ denotes the chemical potential of an oxygen atom and μ_O_  = 0 in the extreme O-rich limit. 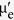 is the corrected chemical potential of electrons, i.e. the Fermi level. L is the linear dimension of the supercell. For simplicity, we employ the value of Madelung constant, 2.8373, corresponding to a simple cubic lattice^36^.

## Additional Information

**How to cite this article**: Li, J. The electronic, structural and magnetic properties of La_1−1/3_Sr_1−3_MnO_3_ film with oxygen vacancy: a first principles investigation. *Sci. Rep.* **6**, 22422; doi: 10.1038/srep22422 (2016).

## Figures and Tables

**Table 1:** Comparison of the structure parameters between the relaxed oxygen-vacancy-containing supercell and the pristine supercell of the same size.

atom-atom distance (Å)	First NN (2 Mn)	Second NN (4 La)	Third NN (8 O)
pristine	3.788	5.585	5.474
oxygen vacancy (GGA)	3.574	5.747	4.973
oxygen vacancy (GGA+U)	3.546	5.743	4.894
**Mn-O-Mn bond angle (**^**°**^)	**a axis**	**b axis**	**c axis**
pristine	180.0^°^	180.0^°^	180.0^°^
oxygen vacancy (GGA)	165.9^°^(177.4^°^)	161.9^°^(173.9^°^)	154.6^°^ (175.1^°^)
oxygen vacancy (GGA+U)	162.1^°^(174.5^°^)	159.3^°^ (169.8^°^)	148.5^°^ (170.2^°^)
**MnO**_**6**_ **octahedron A**	**O–O distance (a axis)**	**O–O distance (b axis)**	**O–O distance (c axis)**
pristine	3.952	3.947	3.788
oxygen vacancy (GGA)	4.054	3.945	3.807
oxygen vacancy (GGA+U)	4.069	3.961	3.822
**Rotated maximum angle with respect to c axis**	11.7^°^ (GGA)	15.7^°^ (GGA+U)	

The atom-atom distance corresponds to the two atoms between which the link passes through the oxygen vacancy. For the Mn-O-Mn bond angle, the values outside the bracket are the minimum angle and inside the bracket are the maximum angle.

**Table 2:** The calculated formation energy (E_f_(O_V_, q) (eV)) for charge state q = −2, −1, +2, +1, and neutral state q = 0.

q	q = −1	q = −2	q = 0	q = +1	q = +2
E_f_(O_V_, q) (eV)	−4.689	−9.988	1.035	7.173	13.722

**Table 3:**
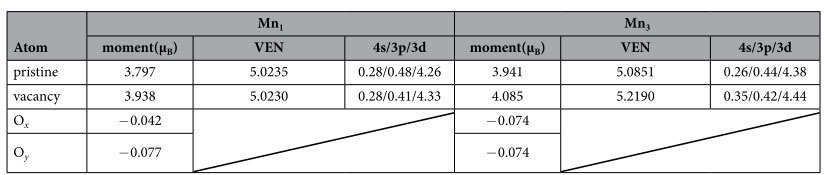
The calculated magnetic moments, total valence electron number (VEN) and valence electron configuration corresponding to the Bader charge analysis of Mn_1_ and Mn_3_ atoms for vacancy and pristine systems.

‘4s/3p/3d’ denotes the valence electron configuration of Mn_1_ and Mn_3_.
